# The Role of Super-Atom Molecular Orbitals in Doped Fullerenes in a Femtosecond Intense Laser Field

**DOI:** 10.1038/s41598-017-00124-9

**Published:** 2017-03-09

**Authors:** Hui Xiong, Benoit Mignolet, Li Fang, Timur Osipov, Thomas J. A. Wolf, Emily Sistrunk, Markus Gühr, Francoise Remacle, Nora Berrah

**Affiliations:** 1 Physics Department, University of Connecticut, Storrs, CT 06269 USA; 2 Departement de Chimie, B6c, Université de Liege, B4000 Liege, Belgium; 3 Center for High Energy Density Science, University of Texas, Austin, TX 78712 USA; 4 LCLS, National Accelerator Laboratory, Menlo Park, CA 94025 USA; 5 Stanford PULSE Institute, SLAC National Accelerator Laboratory, Menlo Park, CA 94025 USA; 6 Institut für Physik und Astronomie, Universität Potsdam, 14476 Potsdam, Germany; 7 Lawrence Livermoor National Laboratory, CA, USA

## Abstract

The interaction of gas phase endohedral fullerene Ho_3_N@C_80_ with intense (0.1–5 × 10^14^ W/cm^2^), short (30 fs), 800 nm laser pulses was investigated. The power law dependence of Ho_3_N@C_80_^q+^, q = 1–2, was found to be different from that of C_60_. Time-dependent density functional theory computations revealed different light-induced ionization mechanisms. Unlike in C_60,_ in doped fullerenes, the breaking of the cage spherical symmetry makes super atomic molecular orbital (SAMO) states optically active. Theoretical calculations suggest that the fast ionization of the SAMO states in Ho_3_N@C_80_ is responsible for the n = 3 power law for singly charged parent molecules at intensities lower than 1.2 × 10^14^ W/cm^2^.

## Introduction

The behavior of atoms, molecules, nano-systems, and solids in intense laser fields^1–14^ continues to reveal new dynamics. Atoms and molecules whose valence electrons can absorb several photons fall in the multiphoton ionization regime class when the Keldysh parameter γ ≫ 1 (γ = (I_p_/2U_p_)^1/2^, where U_p_ is the laser field’s ponderomotive potential and I_p_ is the ionization potential). Those systems whose valence electrons tunnel through the potential barrier formed by the laser field and the Coulomb force, fall within the class of tunneling or over the barrier ionization, depending upon the laser field intensity^5^, and with Keldysh parameter γ ≪ 1^6–10^.

Endohedral, or doped fullerenes, which is the topic of this Scientific Report, are intriguing systems that bridge the gap between molecular and nano-systems^15–18^. However, little is known about their structure and dynamics when excited with a strong laser field. Among their properties, electron transfer from the encaged species to the carbon cage is of special interest^15, 16^. The understanding derived from the photoionization of these carbon nanomaterials enables optimizing their properties, which is relevant to their use in molecular electronics and organic photovoltaics^19^. These nanoscale systems have received attention because they can be used for applications ranging from medical usage^20^ such as in imaging or drug delivery, to their employment in devices for quantum computing^21^. Our interest in investigating these systems stems from the possibility of finding novel fundamental effects making their examination a focus of fundamental research^15, 16, 19, 22^. In fact, we show with this experimental work that doped fullerenes respond differently to intense I.R. laser fields compared to empty fullerenes. Furthermore, with our quantitative calculations, we explain in detail why their behavior is different. This work falls within the general, active topic of non-linear physics^23–33^.

In recent years, non-linear physics or strong-field laser research has led to technological advances and novel phenomena^23–33^. Investigations of the behavior of molecules in short, intense laser fields^5, 10, 34^ were extended to complex molecules, such as C_60_, which has been challenging^35–44^ due to the many electron-nuclei response it exhibits since it is a cage of 60 atoms with 240 valence electrons. The photoionization mechanisms have been found to be wavelength and pulse duration dependent^37, 38, 45^. For IR pulses (800 nm) of about 30 fs duration and intensities below 5 × 10^13^ W/cm^2^, it was found that multiphoton processes dominate when ionizing C_60_, while tunneling, and/or over-the barrier-ionization, or ionization due to induced electron re-collision^36^ are not probable under these conditions. The single-active-electron (SAE) method was used to calculate the ionization of C_60_ in intense, 4 × 10^13^ W/cm^2^, laser pulses with durations between 27 and 70 fs and for a wide range of wavelengths ranging from 395–1800 nm^39^ which agreed with measurements by Shchatsinin *et al.* ^40^. For a long I.R. wavelength of 1800 nm and 70 fs pulse duration, the SAE picture predicts “over the barrier” ionization for a peak intensity of 10^15^ W/cm^2^ leading to non-fragmented but highly charged C_60_^q+^ (q = 1–12)^35^. At short wavelength of 355 nm, the excitation of C_60_ with 10 ns pulses leads to fragmentation by delayed ionization and C_2_ emission as well as other fragments even for small intensities of about 2 × 10^6^ W/cm^2^ ^43^. The use of electron spectroscopy in addition to the ion measurements raised questions for the C_60_ investigations; namely, can the ionization and fragmentation dynamics be adequately modeled in the SAE picture or should multi-electron dynamics be included^40, 44^? This led to recent experimental and theoretical investigation, which concluded that both SAE and many-electron effects, are important^36^.

Our motivation for this work is to contribute substantially to the field of non-linear physics by investigating increased complexity targets. We studied the photoionization of endohedral fullerenes in strong laser fields to answer the following question: Do the ionization dynamics change in fs strong laser fields for a doped fullerene compared to C_60_, an empty fullerene and why? We use a prototype, Ho_3_N@C_80_, interacting with intense (0.1–5 × 10^14^ W/cm^2^), short (30 fs), 800 nm laser pulses giving rise to multiply charged parent ions as well as fragment ions. We focus here on the measurement and theoretical explanation of the power law for singly ionized Ho_3_N@C_80_ yields, and compare our findings to C_60_ results (because pristine C_80_ is not commercially available) carried out under similar conditions^40^. Two distinct regions are present in the ion yield spectra with respect to field strength corresponding to different ionization mechanisms and power laws. For low field strengths, multiphoton ionization dominates, while for higher field strengths, tunneling and ionization over the barrier are the main ionization processes. The photoelectron spectra of endohedral fullerenes such as Li@C_60_^18^ or Sc_3_N@C_80_^46^ have been measured with longer and weaker laser pulses. Although the photoionization of endohedral fullerenes was studied by other groups^13, 14, 18, 46^, none of the cases studied the role of encaged atoms or clusters in femtosecond strong laser fields. As one would expect, encaging of an atom or cluster in a carbon cage modifies the electronic structure of the fullerene, but in what way? Previously, for C_60_, both SAMO and Rydberg states played important roles when exposed to a strong laser field^45^. Both kinds of states can be indirectly populated by vibronic coupling, but because the density of Rydberg states is higher than that of SAMO states, Rydberg state ionization in C_60_ dominates the ionization at laser intensities >10^13^ W/cm^2^ ^45^, which brought up an interesting question: do the SAMO states play a more important role in a doped fullerenes such as Ho_3_N@C_80_? In this work, supported by theory, we demonstrate and explain that the endohedral fullerene Ho_3_N@C_80_ responds differently to intense near-infrared femtosecond laser fields compared to C_60_, because of the increased importance of the SAMO states and this is reflected by a different slope of the power law for low field strengths.

A power law corresponding to n = 5 photon was measured in C_60_ while we report here a power law of n = 3 photons for Ho_3_N@C_80_. In C_60_ the power law is readily understood from energetics: it takes 5 photons of 1.55 eV (800 nm) to reach the IP, 7.6 eV^40^. However, in Ho_3_N@C_80_ (experimental IP ~ 6.9 eV)^44, 45^, instead of observing a power law corresponding to 5 photons, we demonstrate below a power law corresponding to n = 3. This decrease arises from resonance-enhanced multiphoton ionization of intermediate states that promptly ionize by absorption of an extra photon. We argue these states are Super Atomic Molecular Orbital (SAMO) states similar to the ones observed in C_60_^13, 47^. The SAMO states are excited electronic states where a valence electron is promoted to a diffuse atomic-like molecular orbital. They are present in both fullerenes and characterized by very short photoionization lifetimes, in the few fs range, because of their diffuse character^11, 13, 48^. However, unlike in Ho_3_N@C_80_, the lower SAMO states of *s*, *p*, and *d* symmetry are dark states in C_60_ because of their spherical symmetry and cannot be accessed during the pulse. They can only be populated by vibronic coupling on a time scale of few tens of fs. On the contrary, SAMO states of Ho_3_N@C_80_ become optically active because of symmetry breaking induced by the inclusion of Ho_3_N inside the symmetric but non-spherical C_80_ fullerene cage. We show below that the optically active SAMO states in Ho_3_N@C_80_ can be accessed by the absorption of 3 photons during the pulse. Upon absorption of these 3 photons, the SAMO states of Ho_3_N@C_80_ almost instantaneously photoionize, which makes photoexcitation the rate limiting step and explains the n = 3 observed power law. Note that the only theoretically calculated IPs available in the literature are 6.88 eV for Sc_3_N@C_80_^49^ and 6.93 eV for Lu_3_N@C_80_^50^. All of these values are very close to 6.84 eV for the C_80_ molecule^51^.

**Figure 1:**
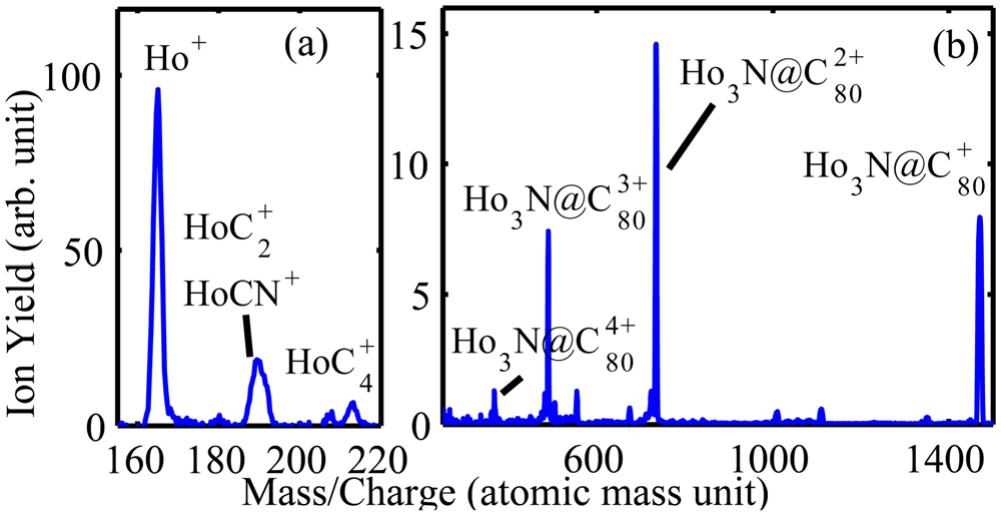
Mass/charge spectra of Ho_3_N@C_80_ ionized with laser pulses of 800 nm, 30 fs pulse duration and an intensity of 4 × 10^14^ W/cm^2^.

**The experiment** was performed using an ion velocity map imaging (VMI) spectrometer used in the time of flight (TOF) mode and details are available in the Supplementary information (S.I.) and in refs 52 and 53. The ion-mass spectrum of Ho_3_N@C_80_ at 800 nm obtained at a laser intensity of 4 × 10^14^ W/cm^2^ and 30 fs pulse duration is shown in Fig. 1. We observe parent ions up to Ho_3_N@C_80_^4+^ although the statistics is low for Ho_3_N@C_80_^4+^. We also observed Ho-based molecular fragment ions: HoC_2_^+^, HoCN^+^, and HoC_4_^+^, as well as atomic Ho^+^ ions (the most abundant) created by bond breaking and bond forming. In this report, we focus on the parent ion yield power laws.

**Figure 2:**
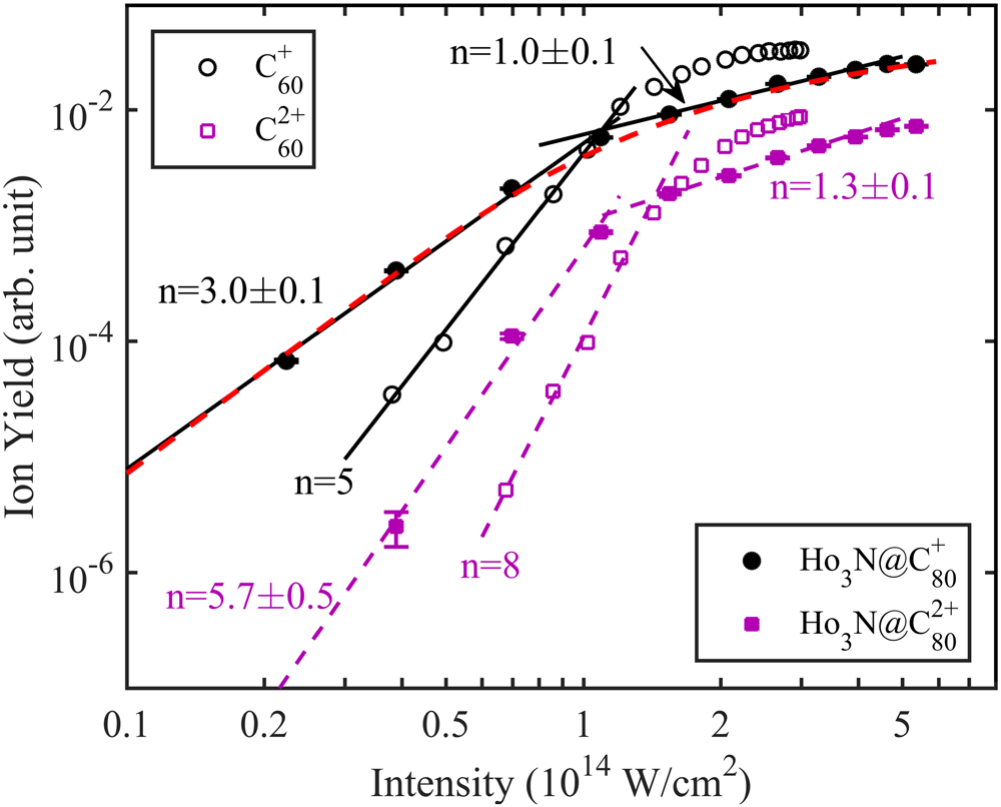
Comparison of the ion yield dependence on peak intensity for the ionization of Ho_3_N@C_80_^q+^ (q = 1, 2) (filled symbols) and C_60_^q+^ (q = 1, 2) (open symbols). The Ho_3_N@C_80_^q+^ results are extracted from data in Fig. 1 and the yields of Ho_3_N@C_80_ with a charge q = 1 is multiplied by 5. The C_60_ data are from ref. 40 and the C_60_^+^ yield was multiplied by 2 for clarity.

**In this work**, we investigated the dependence of Ho_3_N@C_80_^q+^ (q = 1–2) yields as a function of the peak laser intensity and our results are shown in Fig. 2. We find that the yields for Ho_3_N@C_80_ ions follow the power law Y = I^n^ as a function of laser field intensity I^40, 42^. To determine the power laws, linear fits were applied to the experimental ion yields using a least-squares fit for intensities higher than 1.2 × 10^14^ W/cm^2^. Then, the rest of the data, which significantly deviated from the power law at high intensity, were fit by lines with different slopes. The fitting errors for the Ho_3_N@C_80_ ions are shown in Fig. 2. The observed slopes for q = 1, 2 at lower intensities (<1.2 × 10^14^ W/cm^2^) are 3.0 ± 0.1 and 5.7 ± 0.5, respectively and are summarized in Table 1. The intensity at the cross point of the two slopes is defined as the saturation intensity^42^. As the intensity increases beyond saturation for each of the ion species, the yields start to follow an almost linear trend with a shallower slope, indicating that the ionization saturation has been reached^39, 54^.

**Table 1:** Measured power laws for HoN_3_@C_80_ and for C_60_^40^ ion charge states.

Charge state q	q = 1+	q = 2+
C_60_	5	8
Ho_3_N@C_80_	3.0 (±0.1)	5.7 (±0.5)

To check our findings, we performed a simulation of the ion yield of singly charged Ho_3_N@C_80_ through three-photon resonant ionization, taking focal volume averaging into account. The generalized 3-photon cross section σ_3_ is defined by the ionization probability per unit detection volume, W(t) = σ_3_(I/hν)^3^. Experimental parameters used in the simulation are laser focal waist (20 µm), pulse duration (30 fs), and diameter of the molecular beam (4 mm). The yield of singly charged Ho_3_N@C_80_ was obtained by integrating the ionization rate in the interaction region. By adjusting the cross section σ_3_, we obtained a reasonable result shown as the red dashed line in Fig. 2. We note that within the accuracy of the parameters, the result of the simulation does not significantly change.

The strong field ionization (SFI) mechanisms of Ho_3_N@C_80_ with the current IR laser conditions include multiphoton, tunneling, and over the barrier ionization, based on our computation of the barrier lowering induced by the strong field. We find that the multiphoton ionization mechanism dominates at low laser intensities (<5 × 10^13^ W/cm^2^, corresponding to γ > 1.0) and tunnel ionization^5^ dominates at high laser intensities (>10^14^ W/cm^2^, corresponding to γ < 0.75). In the intermediate region, where γ varies between 1.0 and 0.75, there is a complex interplay among the multiphoton, tunneling, over the barrier mechanisms as well as ionization saturation.

In Fig. 2, we compare power law slopes for the doped fullerene results with that of C_60_. The wavelength of the laser used in both experiments was 800 nm, while the laser pulse duration in the C_60_ experiment was 27 fs, close to the 30 fs used in the current experiment and within the error bar. The error bar on the laser intensity was 15%. As can be seen from Table 1, the slopes of power law are substantially higher for the ionization of C_60_ compared to Ho_3_N@C_80_.

**Electronic structure** computations were performed to elucidate the power law difference between C_60_ and Ho_3_N@C_80_. The equilibrium geometry of the Ho_3_N@C_80_’s isomers was computed at the DFT/PBE0^55^ level (see computational details in S.I.). The starting geometries were obtained by adding the Ho_3_N complex inside each of the six C_80_ isomers. The most stable isomer has a slightly triangular C_80_ cage with the nitrogen atom localized at the center, and a Ho-N bond length of 2.06 Å (the shortest compared to the other stable isomers). The inclusion of the Ho_3_N complex in the cage induces a significant lowering of the symmetry. The bare C_80_ belongs to the D_5h_ point group while Ho_3_N@C_80_ exhibits an approximate C_2_ symmetry (see S.I. for details). The computed vertical ionization potential (IP) of the lowest energy isomer is 6.54 eV, which is close to the experimental IP of the C_80_ fullerene (6.84 eV)^51^. The ionization potentials of the other isomers are significantly lower, see Table S1 in the S.I. In the following discussion, we only refer to the lowest energy isomer. Similar doped fullerenes such as Y_3_N@C_80_^56^ and Lu_2_CeN@C_80_^50^ exhibit a triangular cage and a similar ionization potential.

**Figure 3:**
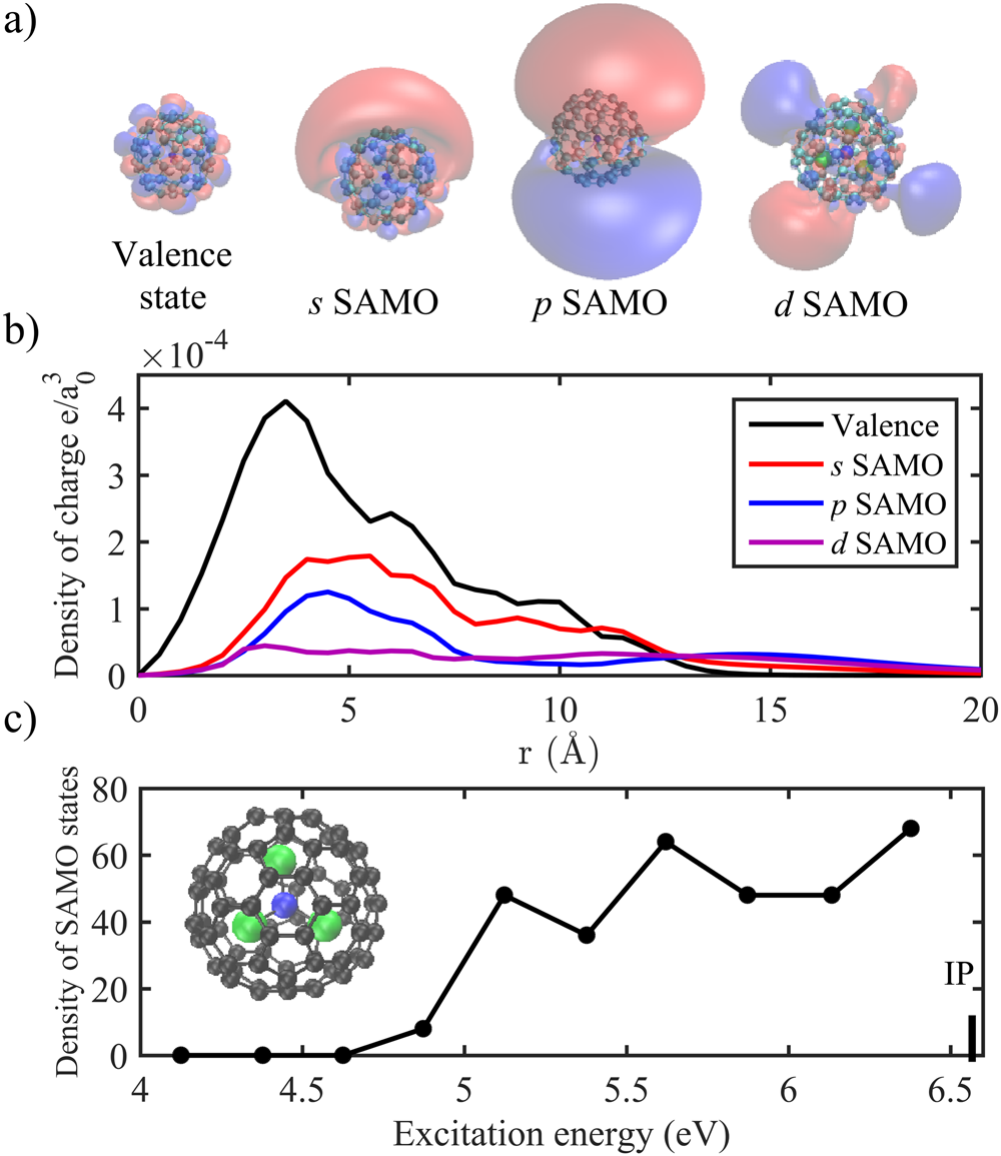
(**a**) Dyson orbitals of a typical valence state, *s* SAMO, *p* SAMO and *d* SAMO states of Ho_3_N@C_80_ with an isocontour of 0.002|e|/Å. (**b**) Charge density of the Dyson orbitals of panel a. (**c**) Density of SAMO states as a function of the excitation energy. The electronic states and Dyson orbitals have been computed in TDDFT at the LC-BLYP/6–31G(d) + ECP56MWB/7s6p5d for Ho + Bq (6–31(6+)G(d)) level for Ho_3_N@C_80_’s lowest energy isomer shown in the inset.

The electronic structures of the 250 lowest SAMO and valence excited electronic states of Ho_3_N@C_80_’s most stable isomer were computed in TDDFT with the LC-BLYP functional^57, 58^ (see computational details in the S.I., note that no Rydberg states are present in the band because their computation requires a larger basis of atomic orbitals that includes more highly diffuse basis functions). Particular attention was given to the excited states coined as super-atom-molecular orbital (SAMO) states^47^. SAMO excited states were first discovered for the case of fullerenes on metal surfaces^47^. They are diffuse hydrogen-like orbitals resulting from the shallow potential present at the center of hollow systems such as fullerenes^11, 13, 14, 47^. The SAMO excited states are different from other Rydberg states because of the significant electronic density localized inside the carbon cage as shown in Fig. 3(a,b). They were called SAMOs because for C_60_, they exhibit the spherical harmonic shapes *s*, *p*, or *d* atomic orbital symmetry^47^. For C_60_ adsorbed on surfaces^47^ and in the gas phase^13, 14^, sharp bands of *s*, *p*, and *d* SAMO’s were identified^48^. Due to the symmetry breaking induced by the insertion of the Ho_3_N inside the cage, in the case of Ho_3_N@C_80_, there is a broad manifold of excited states with delocalized diffuse orbitals of type *s*, *p*, and *d*, analogous to the C_60_’s SAMO states. For this reason, we also named these states SAMO but they are not pure *s*, *p*, or *d* SAMO states as in C_60_ because the presence of Ho_3_N induces a mixing of the SAMO states with the valence states as shown in Fig. 3a. Moreover, unlike those of C_60_, the SAMO states in Ho_3_N@C_80_ are optically active because of the symmetry breaking of the cage. For instance, the transition dipole moment between the ground state and the *s* SAMO state is close to zero in C_60_ and C_80_ while it is 0.66au in Ho_3_N@C_80_ (Table 2).

**Table 2:** Computed binding energies, photoionization lifetimes (in fs) for a field intensity of 10^13^ and 10^14^ W/cm^2^ and transition dipole moments (μ_trans_) between the ground and excited states shown in Fig. 3.

	Excitation energy(eV)	Photoionization lifetime (fs)		μ_trans_ (au)		
		10^13^ W/cm^2^	10^14^ W/cm^2^	Ho_3_N@C_80_	C_60_	C_80_
Valence (ES 67)	4.25	351.03	39.00	1.19	1.55	1.47
*s* SAMO (ES 100)	4.98	2.00	0.22	0.66	0.00	0.02
*p* SAMO (ES 116)	5.23	1.49	0.13	0.69	0.00	0.30
*d* SAMO (ES 145)	5.60	1.56	0.17	0.49	0.00	0.29

The photoionization lifetimes are computed for the ionization of an electron with a kinetic energy of 0.2 eV. For comparison, the computed transition dipole moments of the valence (with an excitation energy around 4.25 eV), *s*, *p* and *d* SAMO states are also given for C_60_ and C_80_.

As can be seen in Fig. 3(c), the number of SAMO states increases with the excitation energy and reaches a maximum at about 5.75 eV. The lowest SAMO state of Ho_3_N@C_80_ has an excitation energy of 4.98 eV and a non-zero transition dipole moment, like all the SAMO states, so that it can be directly accessed from the ground state during the pulse. Its excitation energy corresponds to 3.2 photons but the laser pulse has a FMHW width of 0.15 eV (per photon) so that this state can directly be accessed through 3-photon absorption during the pulse.

Once the SAMO states are photoexcited during the pulse, they can promptly ionize due to their short photoionization lifetime (i.e. the time it takes to photoionize 63% of the population). The lifetimes, that are inversely proportional to the square modulus of the photoionization coupling elements **V**, have been computed for the 250 lowest excited states of Ho_3_N@C_80_ using the formalism described in references^59, 60^.

1 $${\bf{V}}(\varepsilon )={E}_{0}\langle {\varphi }^{Dyson}|{\bf{E}}\cdot {\bf{r}}|{\chi }^{electron}(\varepsilon )\rangle $$

**Figure 4:**
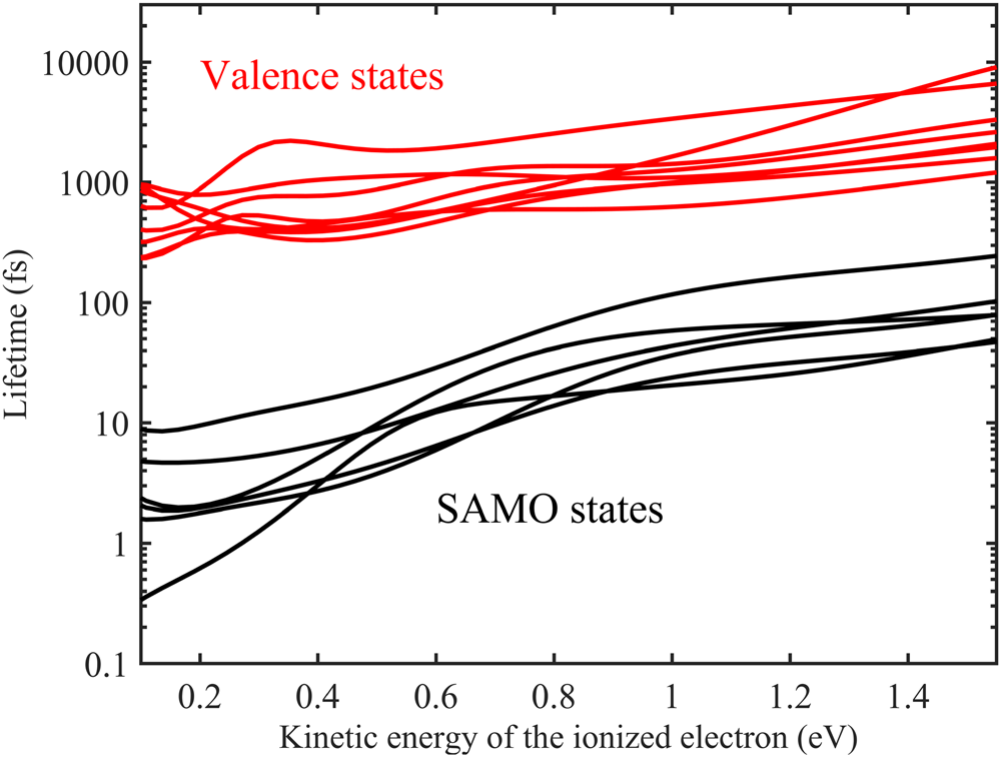
Photoionization lifetime (field intensity of 10^13^ W/cm^2^) for a set of representative SAMO and valence states, plotted as a function of the kinetic energy of electron ionized from the SAMO or valence states. Amongst the valence states, some are isoenergetic with the SAMO states (see S.I. for details).

Here, E_0_ is the electric field amplitude, **E** the electric field polarization vector, χ^electron^ (ε) the wavefunction of the ionized electron and *ϕ* ^*Dyson*^ is the Dyson orbital^11, 48, 61, 62^, which is the overlap between the neutral and cationic wavefunctions. The Dyson orbitals provide a correlated view of the wave function of the ionized electron and play a crucial role in the interpretation of photoelectron lifetimes and angular distributions. We find that the Dyson orbitals of the SAMO states (Fig. 3a) in Ho_3_N@C_80_ have a small valence character and are distorted compared to the ones of the spherical C_60_ fullerene due in part to the loss of symmetry of the slightly triangular C_80_ cage but primarily to the inclusion of Ho_3_N inside the C_80_ cage (Fig. 4a). The presence of Ho_3_N drastically changes the amount of charge density of the Dyson orbital inside the cage compared to that of the bare C_60_. For instance, in C_60_ the charge density of the *s* SAMO is maximal at the center of cage (r = 0) while in Ho_3_N@C_80_, the nitrogen lies at the center of the cage so the *s* SAMO charge density is zero at r = 0 and then increases.

In Eq. 1, the Coulomb interaction between the fullerene cage and the departing electron is neglected, which can affect the momentum of the leaving electron, especially for low kinetic energy electrons. Within this approximation, the wavefunction of the electron is described by an orthogonalized plane wave. Previous work on C_60_ showed that the relative photoionization intensities of SAMO states computed using this approximation were in good agreement with the experimental values^63^, which suggests a minor role of the Coulomb interactions for kinetic energy of the photoelectrons ranging from 0.2 to 1.55 eV. From Eq. 1, we can readily compute the lifetimes that are inversely proportional to the field intensity and to the dipole coupling between the Dyson orbital and the ionized electron. Due to their simple hydrogenoid shape, the SAMO states have a large dipole coupling compared to the isoenergetic valence excited states^48^, which leads to lifetimes several orders of magnitude smaller than the ones of the isoenergetic valence excited states (see Table 2 and Fig. 4(c)). For a field intensity of 10^13^ W/cm^2^, the SAMO states have lifetimes on the order of a femtosecond, which is short compared to several hundreds of fs for the valence states. When the SAMO states are populated, they promptly ionize, unlike the valence states that slowly ionize during the pulse. The limiting rate determining step for the SAMO states is their photoexcitation while for the valence states it is their photoionization. The SAMO state lifetimes strongly depend on the kinetic energy of the ionized electron and increase faster than the valence state lifetimes. Nevertheless, even for a kinetic energy of 1.55 eV (≈one IR photon), the SAMO states still ionize on average 35 times faster than the valence states.

For low laser intensities, the slope of the power law in the photoionization region is expected to be the number of photon necessary to ionize the ground state, as it is the case in C_60_ and which should be 5 for Ho_3_N@C_80_. However, the observed value of “n” is found to be 3 (Table 1), which indicates there are intermediates resonant states^64^ in the photoionization process. These states could be valence or SAMO excited states because they both are optically active (Table 2) and can therefore be transiently populated during the pulse. However, the SAMO states have lifetimes around two orders of magnitude shorter than the valence states so if both type of states are populated during the pulse, ionization would mainly come from the SAMO states. Thus the SAMO states act as fast ionizing intermediate resonant states, which affects the power law, as the rate-determining step is now the photoexcitation process to the SAMO states instead of the photoionization as is the case of C_60_. Such a process does not occur in C_60_ because the SAMO states are not optically active so they cannot be promptly excited from the ground state by direct multiphoton excitation. They can only be populated indirectly by vibronic coupling to isoenergetic optically active valence states^14^, which takes several tens of fs. Therefore, the transient population in the SAMO states during the pulse is larger in Ho_3_N@C_80_ than in C_60_, and so is the ion yield as shown in Fig. 2.

It is known that strong laser fields can induce vibrations in C_60_ ions^65^. Vibrational excitation and significant geometric distortion^66^ may shift the ionization potential of C_60_. However, the laser intensity used in this report is much smaller than the one used in reference^65^. It is thus unlikely that the ionization of the parent molecules was affected by the ionization potential change due to the small vibrational energy accumulated during the laser pulse^65^.

For laser intensities higher than 10^14^ W/cm^2^, where γ < 0.75, ionization over the barrier and tunnel ionization are the main mechanisms. Therefore, the ion yields of Ho_3_N@C_80_ and C_60_ are of the same order because it does not depend anymore on the photoexcitation of the SAMO states during the pulse. In the intermediate region, for field intensity ranging from 5 × 10^13^ − 1 × 10^14^ W/cm^2^, both multiphoton and over the barrier ionization mechanisms coexist. The change of mechanisms is reflected in the ion yield that reaches saturation around 9 × 10^13^ W/cm^2^ for Ho_3_N@C_80_. At this field intensity, we computed a lowering of the IP (second order stark shift) of 1.9 eV, which means that some of the valence states with a slightly higher binding energy than the lowest SAMO states can ionize over the barrier. These states can be accessed by one photon less than the SAMO states and they have transition dipole moments from the ground state up to 5 times larger, which can explain the saturation observed.

**In summary**, this experimental and theoretical work examined the interaction of a complex system, an endohedral fullerene with short, intense laser pulses, revealing fundamental differences compared to the case of an empty cage such as C_60_. We explain quantitatively that the clear signature difference is directly linked to the role of SAMO states in both molecules. Although the dominant photoionization mechanism is multiphoton ionization for both fullerenes, the measurement of the laser intensity power law dependence, I^n^, of the singly and doubly charged Ho_3_N@C_80_ molecule was interestingly found to be different compared to C_60_. This difference was explained in detail for Ho_3_N@C_80_^+^ using TDDFT calculations, revealing that SAMO states act as intermediate resonances with larger photoionization widths compared to isoenergetic valence excited states. Both the C_60_ and Ho_3_N@C_80_ SAMO states have photoionization lifetimes of the order of the fs for field intensity higher than 10^13^ W/cm^2^. The optically active Ho_3_N@C_80_ states can quickly ionize during the 30 fs pulses because they are populated, unlike C_60_’s SAMO states that can only be populated indirectly by vibronic coupling. The SAMO states of Ho_3_N@C_80_ can be optically accessed during the short pulse duration because of their non-zero transition dipole moments, resulting from the symmetry breaking. This experimental and theoretical work demonstrated that the photoionization of a doped fullerene in strong field has a clear signature difference compared to the case of empty C_60_^13, 14^ and of C_60_ adsorbed on surfaces^47^. Furthermore, this work has revealed that an encapsulated molecule inside a cage breaks the fullerene symmetry leading to different photo-dynamics and this result should be general for these nano-systems excited under similar conditions. In addition to contributing new knowledge to the active topic of strong field research, our work on doped fullerenes connects to other areas of science since it impacts the understanding of these nano-systems, which have shown the promise to be used in the design of molecular electronics and optoelectronics materials^47, 67, 68^ as well as used for applications ranging from medical usage^20^ such as in imaging or drug delivery, and in devices for quantum computing^21^.

## Electronic supplementary material

Supplementary Information (S.I.) for The Role of Super-Atom Molecular Orbitals in Doped Fullerenes in a Femtosecond Intense Laser Field
