# Supplementary material for: The Role of Super-Atom Molecular Orbitals in Doped Fullerenes in a Femtosecond Intense Laser Field
Source: Sci Rep. 2017 Mar 9;7:121. doi: 10.1038/s41598-017-00124-9 (PMC5427842; doi:10.1038/s41598-017-00124-9)
Supplement: Supplementary file 1 — Supplementary Information (S.I.) for The Role of Super-Atom Molecular Orbitals in Doped Fullerenes in a Femtosecond Intense Laser Field [file 41598_2017_124_MOESM1_ESM.pdf]

Supplementary Information (S.I.) for

The Role of Super-Atom Molecular Orbitals in Doped Fullerenes in a Femtosecond Intense Laser Field

by Hui Xiong, Benoit Mignolet, Li Fang, Timur Osipov, Thomas J. A. Wolf, Emily Sistrunk, Markus Gühr, Francoise Remacle and Nora Berrah

1) Experimental method

The experiment was performed using an ion velocity map imaging (VMI) spectrometer in time-of-flight (TOF) mode. Details of the spectrometer have been thoroughly described in previous work[1,2]. A mass spectrometer resolution higher than $m/\Delta m = 500$ was achieved for doubly charged $Ho_3N@C_{80}$ with our VMI spectrometer in TOF mode. The sample was evaporated from an oven heated to about 800K. The $Ho_3N@C_{80}$ molecules are injected into the interaction region through an effusive nozzle, where they are intersected by the laser pulses. The ultrashort laser pulses were delivered by a commercially available Ti:Sapphire system. The laser pulses had a duration of 30 fs, measured by a second-harmonic single-shot autocorrelator, and a central wavelength of 800 nm. The laser intensity at the focal spot was varied by an attenuator consisting of a half waveplate and two thin-film polarizers. Two orders of magnitude in intensity (10^{13} – 10^{15} W/cm^2) could be covered by rotating the half-waveplate. The polarization of the laser field was parallel to the spectrometer axis. The laser was focused into the center of the extraction zone by a lens with 35 cm focal length. The focal spot was estimated with the beam diameter and M^2 parameter provided by the manufacturer. The intensity calibration can be achieved by exploiting well-understood nonlinear processes, which in our case is the saturation intensity for the single ionization of Ar[3]. The intensities were determined by scaling the power at the saturation intensity by the power used for the $Ho_3N@C_{80}$ experiments.

2) Electronic structure of the ground state isomers of $Ho_3N@C_{80}$

All the electronic structure computations have been carried out with gaussian09[4]. The equilibrium geometries of the isomers have been obtained with the Perdew, Burke, and Ernzerhof PBE0 [5] functional and the 6-31G(d) basis set for the carbon and nitrogen atoms. For the holmium atom we used a Stuttgart/Cologne effective core potential (ECP) in which 56 of the 67 electrons are included in the multi electron fit quasi relativistic pseudopotential (ECP56MWB)[6,7]. The remaining 11 electrons of the holmium atoms have a 7s6p5d basis set[6].

The equilibrium geometry of the $Ho_3N@C_{80}$'s isomers has been computed by adding the Ho_3N complex inside one of the C_{80} isomers and optimizing the structure at the DFT/PBE0[5] level. From the six isomers of C_{80}[8], five converged (Fig. S1). As stated in the main text, the most stable isomer, #6 in Fig. S1, has a slightly triangular C_{80} cage. Among the four others conformers, two also have a triangular cage (#3 and #4) while the two others have an elongated cage (#1 and #2). The triangular conformers are close in energy to the lowest energy conformer while the two elongated are higher in energy. All the isomers have a longer Ho-N bond length than the lowest energy isomer (Table S1). The vertical ionization potential (IP) of the lowest energy conformer is 6.54 eV, which is close to the experimental IP of the C_{80} fullerene (6.84 eV)[9]. The IP of the other isomers is significantly lower than the one of the lowest energy conformer.

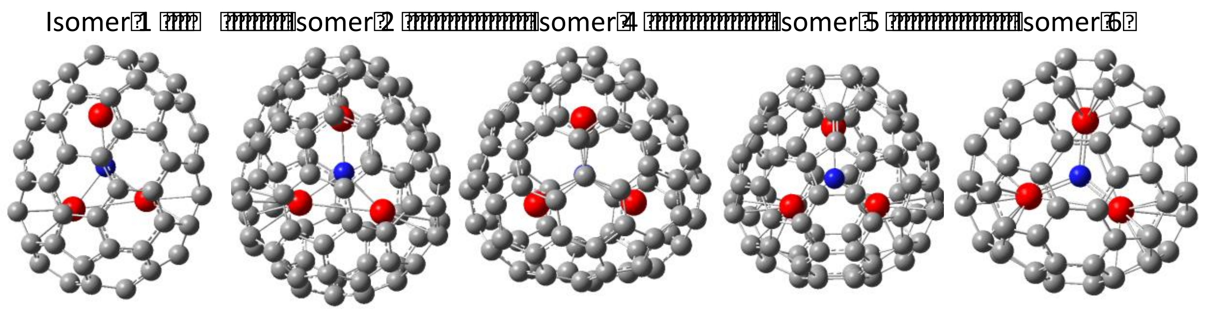

FIG. S1: Equilibrium geometries of the 5 stable isomers of $Ho_3N@C_{80}$. The level of computation is PBE0/6-31G(d)+ ECP56MWB/7s6p5d for Ho atoms.

Table S1: Energy, vertical IP (E_{neut}-E_{cation}) and Ho-N bond length of the $Ho_3N@C_{80}$'s isomers

$Ho_3N@C_{80}$	Isomer #1	Isomer #2	Isomer #4	Isomer #5	Isomer #6
Symmetry of the C_{80} isomer before doping by Ho_3N	D_{5d}	D_2	D_3	C_{2v}	D_{5h}
Approximate symmetry of $Ho_3N@C_{80}$	C_s	C_1	D_3	C_s	C_2
Relative energy to the isomer #6	4.33 eV 100 kcal/mol	2.36 eV 55kcal/mol	0.35 eV 8 kcal/mol	0.89 eV 21 kcal/mol	0.00 eV
Vertical IP (eV)	5.85	5.75	6.03	5.93	6.54
Ho-N bond length (Å)	2.09, 2.11, 2.14	2.11, 2.11, 2.15	2.15, 2.15, 2.15	2.07, 2.07, 2.08	2.05, 2.05, 2.06

3) Electronic structure of the excited states of the lowest energy isomer

The electronic structure of the 250 lowest excited states of $Ho_3N@C_{80}$'s have been computed with the linear implementation of TDDFT with the Long-range Corrected Becke Lee Yang and Parr functional[10,11] (LC-BLYP) with an optimized long-range parameter[12] (ω=0.17), the same basis set as for the optimization augmented by 6 diffuse *s*, *p* and *d* basis functions centered in the middle of the cage[13]. The number of excitations allowed to build the excited states is restricted. Only excitations from the five highest occupied orbitals to the 65 lowest virtual orbitals are allowed. Because the SAMO states are mainly composed of excitations from the HOMO to several virtual SAMO molecular orbitals, reducing the excitation space does not have significant consequences on the binding energies of the SAMO states but allows reducing significantly the computer time needed for these calculations[14]. Amongst these excited states, we differentiate the SAMO states from the other excited states that are called valence states. It should be noted than unlike in reference[14], there are no Rydberg states amongst the excited states because the basis used for the computation of the electronic structure does not contain very diffuse atomic orbitals. The IP in LC-BLYP with basis set containing extra diffuse function is 6.55 eV, which is close to the IP computed in PBE0 (6.54 eV) and the experimental IP of C_{80} (6.84 eV).

The electronic structure of the excited states and their photoionization lifetimes have also been computed with the CAM-B3LYP functional and the same basis than the one used in LC-BLYP. The energy is the first SAMO is 0.4 eV higher than in LC-BLYP and the photoionization lifetimes of the SAMO and valence states are in the same order of magnitude.

References

1 Pešić, Z. D., Rolles, D., Dumitriu, I. & Berrah, N. Fragmentation dynamics of gas-phase furan following K-shell ionization. *Phys. Rev. A* **82**, 013401, (2010).
2 Berrah, N. *et al.* Femtosecond X-ray-induced fragmentation of fullerenes. *J. Mod. Opt.* **63**, 390-401, (2016).
3 Hankin, S. M., Villeneuve, D. M., Corkum, P. B. & Rayner, D. M. Intense-field laser ionization rates in atoms and molecules. *Phys. Rev. A* **64**, 013405, (2001).
4 Frisch, M. *et al.* Gaussian 09 (Revision C), Gauussian Inc., Wallingford CT (2009).
5 Adamo, C. & Barone, V. Toward reliable density functional methods without adjustable parameters: The PBE0 model. *J. Chem. Phys.* **110**, 6158-6170, (1999).
6 Dolg, M., Stoll, H., Savin, A. & Preuss, H. Energy-adjusted pseudopotentials for the rare earth elements. *Theor. Chim. Acta* **75**, 173-194, (1989).
7 Dolg, M., Stoll, H. & Preuss, H. A combination of quasirelativistic pseudopotential and ligand field calculations for lanthanoid compounds. *Theor. Chim. Acta* **85**, 441-450, (1993).
8 http://www.nanotube.msu.edu/fullerene/fullerene.php?C=80.
9 Zimmerman, J. A., Eyler, J. R., Bach, S. B. H. & McElvany, S. W. ‘‘Magic number’’ carbon clusters: Ionization potentials and selective reactivity. *J. Chem. Phys.* **94**, 3556-3562, (1991).
10 Becke, A. D. Density-functional exchange-energy approximation with correct asymptotic behavior. *Phys. Rev. A* **38**, 3098-3100, (1988).
11 Iikura, H., Tsuneda, T., Yanai, T. & Hirao, K. A long-range correction scheme for generalized-gradient-approximation exchange functionals. *J. Chem. Phys.* **115**, 3540-3544, (2001).
12 Zhang, C.-R. *et al.* Theoretical Study of the Local and Charge-Transfer Excitations in Model Complexes of Pentacene-C_{60} Using Tuned Range-Separated Hybrid Functionals. *J. Chem. Theory Comput.* **10**, 2379-2388, (2014).
13 Mignolet, B., Johansson, J. O., Campbell, E. E. B. & Remacle, F. Probing Rapidly-Ionizing Super-Atom Molecular Orbitals in C_{60}: A Computational and Femtosecond Photoelectron Spectroscopy Study. *ChemPhysChem* **14**, 3332-3340, (2013).
14 Mignolet, B. & Remacle, F. AIP conference proceedings, in press. (2016).
